# A Reality Checkpoint for Mobile Health: Three Challenges to Overcome

**DOI:** 10.1371/journal.pmed.1001395

**Published:** 2013-02-26

**Authors:** The PLOS Medicine Editors

The use of mobile electronic devices to support medical or public health practice, or m-health, is currently a hot topic. It has been predicted that by 2017 there will be “more mobile phones than people” on the planet [1], and currently three-quarters of the world's population have access to a mobile phone [2]. The World Health Organization (WHO) has announced [3] that m-health has the “potential to transform the face of health service delivery across the globe,” and there is increasing media and consumer interest in m-health, illustrated for example by a recent panel at the US Consumer Electronics Show on “The Digital Health Revolution” [4]. Survey data illustrates that most regions of the world, including many low- and middle-income countries, are actively working on m-health pilot projects or have set up systems, for example, for managing treatment compliance, sending appointment reminders, or conducting surveys [3].

However, amidst the interest (and, possibly, a bit of hype) it is worth considering whether m-health needs a reality check. Recently in *PLOS Medicine*, an Essay by Mark Tomlinson and colleagues [5] highlighted the proliferation of m-health pilots in many countries. However, in the Essay, Tomlinson and colleagues comment that few pilots move forward to scale-up, and there is little evidence to inform whether, when, and how, pilots might expand countrywide. Tomlinson and colleagues also raise concerns regarding the increasing interest in m-health from industry, which is likely to have very different motivations than would patients or those responsible for safeguarding public health.

At a WHO forum on data standards for e-health (defined as the use of electronic processes and communication to support health care), held in December 2012 [6] (where one of us, EV, participated), countries reported that a panoply of proliferating standards exists, many of which are closed standards, with high barriers to access; barriers include not just cost, but also the technical complexity of systems and standards and language differences. Consequently, in the rush to develop new applications, many countries end up with a fragmented patchwork of systems, which do not talk to each other. Although these concerns were raised in the context of e-health, the same issues also apply to m-health. With this in mind, we set out three key challenges that advocates will need to overcome to fulfill the promise of m-health.

## Reality Check 1: Are Your Systems Interoperable?

Interoperability refers to those properties of systems (whether software, communications, or other systems), that enable the exchange of data among systems in common formats, the use of common protocols, and ultimately the ability to work together. Interoperability is a critical issue for m-health (and for e-health more generally), because patients may have multiple clinical needs and conditions at one time, and will interact with the health systems via multiple points, providers, and professionals [7]. Although many m-health applications may appear simple (e.g., a system sending texts to patients reminding them of their next appointment), such systems will have greatest potential for wider use if they can easily and accurately exchange information with other systems—for example, microscopy images taken using a mobile phone can be securely imported back into the electronic health record.

A vision for interoperability [8] has set out what should live inside the interoperable “core” of m-health systems: standards that govern health data concepts, patient identity, data processing protocols, and mechanisms for secure sharing of patient data that preserve confidentiality. However, while common standards for all these uses do not yet exist, there is light at the end of the tunnel. As Estrin and Sim have emphasized [8], there are critical differences between the e-health and m-health fields. M-health has fewer entrenched systems than does e-health, and therefore potentially fewer legacy barriers to overcome in order to establish a new shared architecture.

## Reality Check 2: Are You Using Open Standards?

Open standards and interoperability go hand in hand, although these terms refer to different properties of a system [8]. There is no single definition of “open standard,” but generally this term is taken to imply that the standard is publicly available, information about its use and application is available, there are no fees for use of the standard, and the standard was developed using a consensus process [9]. Multiple “standards” exist in e- and m-health—for example, Health Level Seven (HL7) refers to a set of rules that govern how health-care systems exchange information with each other. SNOMED CT is a coded taxonomy which is used to define healthcare information concepts (e.g., to define diseases, findings, procedures, and so on. However, although both examples have been developed by nonprofit organizations, neither is yet freely available for use (although HL7 hopes to soon make its standards free [10]). Development of open standards is particularly critical for equity in e- and m-health. Contributors to the WHO forum on data standards [6] emphasized that closed standards create a knowledge barrier for systems developers in low- and middle-income countries. Without access to the standard, programmers and policy makers cannot understand how the systems or standards work, cannot develop technical capacity, and ultimately cannot make good decisions for their country about what systems they need to develop and how. There is a need for a governing body—for example, WHO—to certify open standards and enable countries' access to standards that meet key criteria.

## Reality Check 3: How Will You Evaluate?

Finally, a key challenge for the m-health field relates to evaluation—not just the need for better and more evaluation, but also the challenge of knowing how to evaluate. In January 2013 *PLOS Medicine* published two systematic reviews of randomized trials in m-health addressing the use of such interventions to improve health behaviors, improve disease self-management, and facilitate health-care delivery processes [11],[12]. Caroline Free and colleagues found that while many studies have been conducted, many are of poor quality, few have low risk of bias, and very few have found clinically significant benefits of the interventions. However, analyses do highlight some evidence suggesting benefits from specific m-health applications, such as those helping patients quit smoking, improving HIV medication adherence, and modestly improving aspects of clinical diagnosis and management. The authors emphasize, crucially, that high-quality trials are needed to provide a much more rigorous evidence base to inform scale-up of m-health applications. Furthermore, the authors found very few studies that had been conducted in low- and middle-income countries, which are needed to evaluate feasibility and efficacy of m-health in such settings, including comparing interventions with control groups that reflect real-world conditions. However, some viewpoints suggest that focusing exclusively on randomized trials as the “gold standard” for evaluating interventions is too restrictive for m-health [13]. After all, m-health applications can best be thought of as complex interventions—because to be effective, such interventions will entail changes to the behavior of health-care professionals or patients who will use them, and potentially may also involve changes to systems or processes involved in delivery of care. Some researchers [13],[14],[15] have suggested that complex interventions require a broader definition of what constitutes evidence—for example, suggesting that theoretical and qualitative approaches are necessary to help policy makers go beyond evaluating *whether* an m-health application works to understand *why, what, and under what conditions* it works (or does not work). Standards are currently being developed [15] for such approaches, termed “realist” or “meta-narrative” methods. M-health aficionados would do well to follow the emerging debate in this area and avoid taking too narrow a view of what constitutes “evidence” in this fast-moving field.

Given the challenges for m-health, cooperation and coordination between very different stakeholders will be necessary to deliver on its potential.

## References

[1] Garside J (06 June 2012) More mobile devices than people ‘within five years.” Available: http://www.guardian.co.uk/business/2012/jun/06/more-mobile-devices-people-five-years. Accessed 15 January 2013.

[2] [No author listed] (17 July 2012) Mobile phone access reaches three quarters of planet's population. Available: http://www.worldbank.org/en/news/2012/07/17/mobile-phone-access-reaches-three-quarters-planets-population. Accessed 15 January 2013.

[3] WHO Global Observatory for eHealth (2011) New horizons for health through mobile technologies. Geneva: World Health Organization. Available: http://www.who.int/goe/publications/ehealth_series_vol3/en/. Accessed 15 January 2013.

[4] Huffington A (07 January 2013) CES 2013, GPS for the soul and the digital health revolution. Available: http://www.huffingtonpost.com/arianna-huffington/ces-gps-for-the-soul-app_b_2426569.html?utm_source=DailyBrief&utm_campaign=010813&utm_medium=email&utm_content=FeatureTitle&utm_term=Daily%20Brief. Accessed 15 January 2013.

[5] Tomlinson M, Rotheram-Borus MJ, Swartz L, Tsai AC (2013) Scaling Up mHealth: Where is the evidence? PLoS Med 10 (2) e1001382 doi:10.1371/journal.pmed.1001382. 23424286 10.1371/journal.pmed.1001382 PMC3570540

[6] WHO (2013) Call for innovative health technologies. Available: http://www.who.int/ehealth/en/. Accessed 15 January 2013.

[7] van Heerden A, Tomlinson M, Swartz L (2012) Point of care in your pocket: a research agenda for the field of m-health. Bull World Health Organ 90: 393–394 doi:10.2471/BLT.11.099788. 22589575 10.2471/BLT.11.099788 PMC3341694

[8] Estrin D, Sim I (2010) Open mHealth architecture: an engine for health care innovation. Science 350: 759–760. 10.1126/science.1196187 21051617

[9] [No author listed] (2012) Open standard. Available: http://en.wikipedia.org/wiki/Open_standard. Accessed 15 January 2013.

[10] Mearian L (04 September 2012) HL7 e-health information-sharing standard to be free. Available: http://www.computerworld.com/s/article/9230896/HL7_e_health_information_sharing_standard_to_be_free?source=CTWNLE_nlt_ithealthcare_2012-09-05. Accessed 15 January 2012.

[11] Free C, Phillips G, Watson L, Galli L, Felix L, et al (2013) The Effectiveness of Mobile-Health Technologies to Improve Health Care Service Delivery Processes: A Systematic Review and Meta-Analysis. PLoS Med 10 (1) e1001363 doi:10.1371/journal.pmed.1001363. 23458994 10.1371/journal.pmed.1001363 PMC3566926

[12] Free C, Phillips G, Galli L, Watson L, Felix L, et al (2013) The Effectiveness of Mobile-Health Technology-Based Health Behaviour Change or Disease Management Interventions for Health Care Consumers: A Systematic Review. PLoS Med 10 (1) e1001362 doi:10.1371/journal.pmed.1001362. 23349621 10.1371/journal.pmed.1001362 PMC3548655

[13] Walshe K (2007) Understanding what works – and why – in quality improvement – the need for theory driven evaluation. Int J Qual Health Care 19: 57–59. 17337518 10.1093/intqhc/mzm004

[14] Shepperd S, Lewin S, Straus S, Clarke M, Eccles MP, et al (2009) Can We Systematically Review Studies That Evaluate Complex Interventions? PLoS Med 6 (8) e1000086 doi:10.1371/journal.pmed.1000086. 19668360 10.1371/journal.pmed.1000086 PMC2717209

[15] Greenhalgh T, Wong G, Westhorp G, Pawson R (2011) Protocol - realist and meta-narrative evidence synthesis: Evolving Standards (RAMESES). BMC Med Res Methodol 11: 115 doi:10.1186/1471-2288-11-115. 21843376 10.1186/1471-2288-11-115 PMC3173389

